# Knowledge, skills and attitudes of older people and staff about getting up from the floor following a fall: a qualitative investigation

**DOI:** 10.1186/s12877-020-01790-7

**Published:** 2020-10-06

**Authors:** Dawn R. Swancutt, Suzy V. Hope, Benjamin P. Kent, Maria Robinson, Victoria A. Goodwin

**Affiliations:** 1 University of Plymouth, Plymouth, England; 2 2.05D South Cloisters, St Luke’s Campus, Magdalen Road, Exeter, EX1 2LU England; 3 Royal Devon and Exeter NHS Foundation Trust, Exeter, England; 4 South Western Ambulance Service NHS Foundation Trust, Exeter, England

**Keywords:** Falls, Rehabilitation, Qualitative, Older people, Capability, Getting up

## Abstract

**Background:**

Falls are the most common reason for ambulance callouts resulting in non-conveyance. Even in the absence of injury, only half of those who fall can get themselves up off the floor, often remaining there over an hour, increasing risk of complications.

There are recognized techniques for older people to learn how to get up after a fall, but these are rarely taught. The aim of this study was to develop an understanding of attitudes of older people, carers and health professionals on getting up following a fall.

**Methods:**

A qualitative focus group and semi-structured interviews were conducted with 28 participants, including community-dwelling older people with experience of a non-injurious fall, carers, physiotherapists, occupational therapists, paramedics and first responders. Data were transcribed and analysed systematically using the Framework approach. A stakeholder group of falls experts and service users advised during analysis.

**Results:**

The data highlighted three areas contributing to an individual’s capability to get-up following a fall: the environment (physical and social); physical ability; and degree of self-efficacy (attitude and beliefs about their own ability). These factors fell within the wider context of making a decision about needing help, which included what training and knowledge each person already had to manage their fall response.

Staff described how they balance their responsibilities, prioritising the individual’s immediate needs; this leaves limited time to address capability in the aforementioned three areas. Paramedics, routinely responding to falls, only receive training on getting-up techniques from within their peer-group. Therapists are aware of the skillset to breakdown the getting-up process, but, with limited time, select who to teach these techniques to.

**Conclusion:**

Neither therapists nor ambulance service staff routinely teach strategies on how to get up, meaning that healthcare professionals largely have a reactive role in managing falls. Interventions that address the environment, physical ability and self-efficacy could positively impact on peoples’ capability to get up following a fall. Therefore, a more proactive approach would be to teach people techniques to manage these aspects of future falls and to provide them easily accessible information.

## Introduction

### Background

Falls are common, affecting a third of people over 65, and half of those aged over 80 each year [1]. Despite some evidence of effective interventions to reduce falls among older people [2–4], most falls are not prevented and we must consider how we can best meet the needs of those who continue to fall, whilst bearing in mind the cost-effectiveness of interventions and implications for service delivery.

Falls are the largest driver of ambulance service demand [5, 6]. It is estimated that approximately half of people who fall without injury are able to get themselves off the floor [7]. Living alone, reduced mobility and cognitive impairment are factors associated with in in-ability to get up from a fall [8]. Of the falls-related calls to emergency services, 11–56% do not result in conveyance to hospital [9]. Most people unable to get themselves up after a fall remain on the floor for more than an hour [8], thus increasing the risk of complications, such as pressure sores, pneumonia and even death. It is not only the person who has fallen who suffers; carers suffer distress due to the person fallen not being able to get up, or not being able to help them. They may be unsure how best to assist, which may result in their own injury or the need for additional assistance [10, 11].

UK national recommendations are that verbal and written advice and practical sessions be provided to older people on how to cope with a fall, and avoid a long lie on the floor [12]. However, data reveals limited implementation of this guidance. One UK survey found that practical demonstrations were reported by only 14/231 (6%) of falls services [13], and UK audit data found in 2010 that only 4% of fallers in rehabilitation programmes were taught how to get up [14]. Anecdotally there appears to be some reluctance to teaching techniques for getting up off the floor - possibly due to a lack of confidence from both older people and therapists, although there is evidence that it is feasible and acceptable to teach this to inpatients, community-dwelling older people and stroke survivors [15–17]. One method commonly used, called “backward chaining” breaks the activity of getting down to and up from the floor into smaller steps, with the last step being taught and achieved first [18].

Interventions that could change the nature of individuals’ response to a fall could be beneficial in terms of improving personal independence and reducing use of NHS resources [8]. Yet, despite the large body of evidence focusing on preventing falls, there is a paucity of research understanding attitudes of older people, carers and healthcare professionals about getting up from a fall [17].

### Aims and objectives

The aim of this study was to develop an understanding of attitudes towards seeking help and using and teaching techniques to get up following a fall.

The objectives were to:

- Interview older people who have experienced a fall, and their carers to establish what actions they take when they fall, and, how and why they make those decisions
- Interview healthcare providers to establish how they support older people in managing future falls, and what factors influence their actions.

## Methods

A qualitative study design was used to explore the knowledge, skills and attitudes of older people and carers following a fall, and to understand how ambulance and rehabilitation staff provide support to older people when dealing with a fall.

### Sampling and recruitment

For those affected by falls personally, we employed a purposive sampling strategy to achieve a range in participant age, sex, living arrangements and co-morbidities. Inclusion criteria were for people aged 65 and over, living in Devon, with experience of a fall and inability to get up (with or without injury). Participants were identified from: (a) Older adults/carers who called an ambulance following a fall without requiring conveyance to hospital; (b) Older people (and carers) attending a local falls service*.* They were offered study information by their therapist or paramedic. If interested, participants completed a consent to contact form and were phoned and then visited by the researcher (DS). The researcher (DS) held no pre-existing views about the falls services or individuals response to falling.

We aimed to interview healthcare professionals including physiotherapists and occupational therapists working in acute hospital, outpatient or home-based rehabilitation settings (including falls services), and ambulance service staff including paramedics, first responders and control room staff. All had to have direct experience of working with older people who had fallen. These staff groups were chosen as they are the professionals most likely to be involved in advising, assisting or teaching someone to get up after a fall. Health professional participants were approached to participate in the study by newsletter and word of mouth.

### Data collection

Face-to-face and semi structured interviews, following a topic guide (see Additional File 1), were conducted with the participants who had experience of a fall. If the participant’s spouse was present, they were also invited to contribute to the interview. Interviews were conducted at the participant’s home and lasted up to 1 h.

Two 90 min focus groups were planned for healthcare professionals involved in the care or rehabilitation of people following a fall (therapists and ambulance service staff). The ambulance service staff focus group was adjusted to face-to-face interviews to accommodate differences in shift working patterns and practical challenges in getting people together. One researcher (DS) conducted the interviews. DS and VG facilitated the focus group. The researchers collecting data were female, working in health and health research, DS is experienced in qualitative investigation and trained in Framework analysis.

The topic guide used for older people was developed in conjunction with public and patient involvement representatives, and the topic guide for healthcare professionals also included expert clinical input. All data were audio recorded and underwent targeted transcription to facilitate rapid identification of relevant issues for refining findings. Field notes were taken during the interviews and focus group. Data collection continued until saturation occurred, meaning that no new ideas were being generated [19].

### Analysis

The data analysis was structured using the Framework approach to systematically analyse the data [20]. This method is recognised for its applicability to applied health research and multidisciplinary team working. Transcripts were read independently by two researchers, a coding framework was developed to represent the main themes in the data. Data analysis took a critical realist perspective and focussed on understanding the causal mechanisms underlying decisions made by older people and carers on what action they take when they fall, and understanding what healthcare professionals do that may impact on those decisions.

The transcripts were then double coded by members of the research team to ensure applicability of the coding framework (DS, VG, SH, BK). Where disagreement arose the coding was discussed and resolved. Coded data was charted into the framework matrix (spreadsheet format). Within and between-case analysis was conducted to develop theoretical concepts from the initial coded categories, expanding on how initial actions and choices could be described conceptually. The wider research team and the stakeholder advisory group contributed to the interpretation of the findings. The stakeholder advisory group comprised three specialists in falls rehabilitation, three people with experience of falls and a carer with experience of helping people who fall. They supported the analysis with feedback on the meaning ascribed to the findings and as an independent member check to ensure the quality of analysis.

## Results

In total, 28 older people, spouses and healthcare professionals participated in the study; sixteen interviews were conducted with older people including four spouses, and twelve healthcare professionals participated in either a focus group (*n* = 7) or interview (*n* = 5) about their experience of managing those who fall. Only one potential participant declined to be interviewed due to poor health on the day. Participant characteristics are detailed in Table 1. All participants with experience of having a fall were recruited via falls rehabilitation services and outpatient clinics, and none through the ambulance service. All participants had more than one fall. The majority of participants chose not to call an ambulance as a response to their fall, but did need help to get up, the minority had an ambulance called for them and experienced non-conveyance.

**Table 1:** Participant characteristics

**Type of participant – Older people**	**Number**	**Sex** **M:F**
Older people with experience of having a fall	12	5:7
Spouse of participants	4	1:3
Living arrangements:		
Lives with spouse	7	
Lives alone	3	
Lives alone in sheltered housing	2	
Age range of participants (average)	65-89 (78)	
**Type of participant – Healthcare staff**	**Number**	**Sex** **M:F**
Paramedic staff	5	3:2
Therapy staff (occupational and physio)	7	2:5

**Fig. 1:**
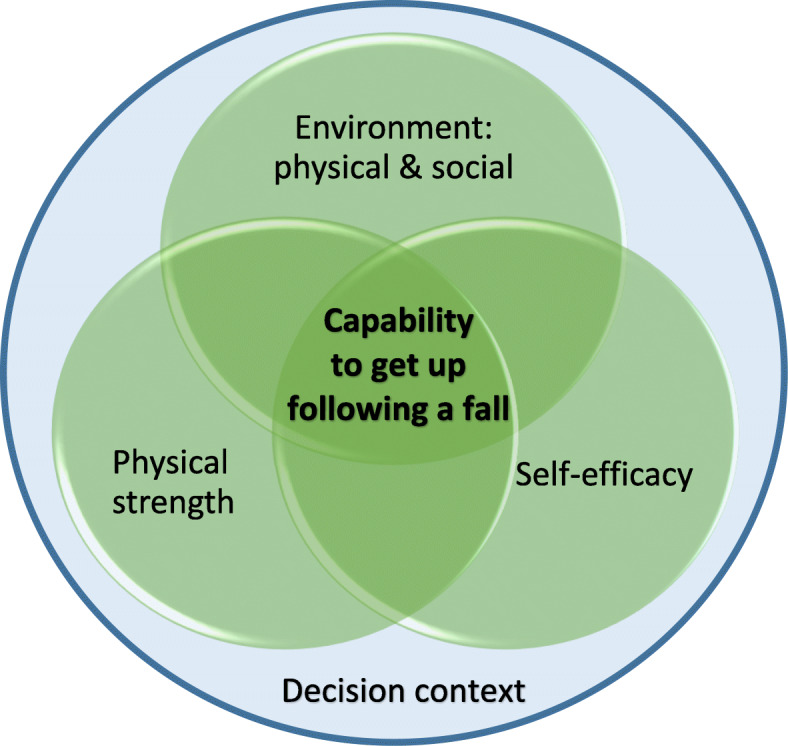
Evidence-based model of potential interventions to enhance capability to get up following a fall

Following the stakeholder advisory group review of analysis, the initial coding themes were developed. Five main themes emerged from the data: personal preventative strategies (including their environment, activities and training on getting up), personal reactive actions (including social network, formal services and incidental contacts), confidence (personal physical/practical and fear, or lack of), support services (therapists, balance groups, other linked services) and independence (in daily life, in physical ability, personal risk assessment and perceptions of others). From these themes we created a model of capability, comprising three main components of an individual’s perceived capability to manage the response to a fall themselves within a decision context (Fig. 1). Although components were distinct, they impacted closely upon each other, for example, physical capability in one environment may differ in another where adaptions were available. ‘Having the know-how’ and ‘a balancing act’ contributed to the decision context. We present these components below, giving the older person and staff views together to form a holistic picture of knowledge, skills and attitudes.

### The environment

People commonly began by describing their immediate environment following a fall, including both the physical and the social environments. Whether they were indoors or outdoors was viewed as significant, particularly in relation to what stable objects were nearby that might be used to ‘pull’ oneself up. Outdoors was viewed as more challenging as often an open space with no objects near and where no-one might pass to find them and offer help.

*“I’ve always had someone available to help me out and so, that hasn’t been a problem as yet but if I was to fall outside I am certain I’d have to call for someone to help me”* Participant #1

*“If we can get him onto all fours and then if there is a chair nearby and he can sort of push himself up on the chair as well as me lifting [wife]”* Participant #2 spouse

The social setting, both immediate (who was with them or near enough to hear them), and wider (who they could call easily, or might drop by), gave a sense of safety and security, whether they chose to draw upon that resource or not.

*“I was very fortunate ‘cos my grandson called in, he calls in quite a bit and makes a sandwich or something you know and he said ‘hi Nana’ I said ‘can you come up here and help me’ and he got me up but it was a struggle”* Participant #4

*“I know there’s benches there [on that walk] and then there’s the low walls that way. I sort of go in my mind what’s where. ... So, if I did have a fall, I’m not saying it’s going to be in that precise, but if I did, I knew I could help myself but there’s not always people about”* Participant #5

Many reported not wanting to ‘bother’ anyone.

*“Well I suppose if you have passed out then you would think that perhaps something was untoward and you needed to do something … . The victim is always reluctant oh don’t make a fuss but obviously the onlooker knows that there’s a problem.”* Participant #3

One Paramedic summed up what many of our older participants said.

“*The person who’s fallen quite often is embarrassed to have to call … that’s what I’ve found so far, is, elderly generation, they don’t want to be a bother … . they just want to get up.”* Ambulance service staff #5

During the therapist focus group a view was raised about the impact of tailoring information to a person’s environment to make it more applicable. This was a common adaptation within the therapist profession.

*“Giving generic advice to people in clinic isn’t that helpful if it doesn’t relate to their situation so seeing people in their homes and being able to adapt to their environment and their needs and their presentation is where I feel we make the biggest impact.”* Therapist focus group

### Physical ability

Deteriorating physical ability was a common theme amongst all participants, this was especially noticed in those with long term conditions such as Parkinson’s. Some described a lack of physical strength hampering the ability to move from the position of the fall. Where the individual had enough strength to move to a better position or location (including with assistance from another person), some could identify a situation from which they made the most effective use of the strength they had, such as shuffling or crawling to stairs indoors or a tree outdoors to use more of their upper body strength, rather than legs alone, illustrating the relationship between strength and environment.

*“most of the time I have had the strength, it’s only in the last couple of years now that I don’t have the strength in my, once my knees get below a certain point they won’t take my weight so I can’t get up above that point to do it now”* Participant #9

“*I think once or twice you’ve fallen and you’ve got your, you’ve crawled to a tree and pulled yourself up holding onto a tree but which you can probably still do now I don’t know. Maybe not [wife]” Participant #2 spouse*

In the therapist focus group only one type of getting up technique was mentioned, called backward chaining – a process of breaking down the tasks needed to be able to get up into a series of movements and practising those movements from a position of safety. A belief was aired that only people most likely to benefit from learning these skills would be taught them; individuals would need enough strength to manage the technique and be willing to practise (with coaching and encouragement). However, one participant described how training can occur even if the person does not initially have the strength:

*“In certain patients, maybe see them for a month or so, and try and build them back up a little bit if they haven’t had much exercise for a while, and then they might reach a point where they will be able to [learn backward chaining techniques].”* Therapist focus group

This “skilling up” approach not only addressed physical strength but also supported the final concept of self-efficacy. Older people corroborated this strength building ability.

*“[therapist name] says now that my legs are weak and I’ve got to try and strengthen that so, that’s what I am doing now I am going to exercise classes on a Tuesday morning”* Participant #3

*“The physio has been helping me a little bit... With me getting up and getting down. Before I used to just, I’d have to drop down and I’ve got more strength in my leg now it’s sort of helping me. Like in the house if I have a fall in the house, I can lift myself up better”* Participant #5

### Self-efficacy

The concept of self-efficacy, ie attitude and beliefs about their own ability came through strongly, both with positive and negative beliefs. This concept generally seemed to originate from what seemed to be the psychological reaction to falling, building from the initial shock of the fall, to a longer-term fear of falling and ‘activity avoidance’ therefore limiting personal exposure to risky activities or locations. Often people couldn’t verbalise exactly what prevented them from learning getting up techniques.

*“I do find it difficult to get up. I do worry about it, especially, if I am on my own... I am a bit funny about this getting up business because in autumn … I went to balance classes … they were prepared to show any of us how to get up you know from the floor. I didn’t want to do it, it was funny really... but I am funny about those sorts of things. I am trying not to fall now anyway that’s the main thing. [why didn't want to try learning to get up?] I am not sure to be honest I know there was an elderly gentleman he was about my age and he seemed very frail, but he went through it with the physio...which I thought was wonderful.”* Participant #5

However one participant expressed an alternative view, of frustration rather than fear. Thus it was not only the psychological impact of being fearful, but also their personal self-efficacy and confidence in their own ability.

*“But not [send an ambulance] just because I’m on the floor. I know I’m on the floor and I can’t get up and it’s really annoying, and if you are down there for three hours it’s very, very annoying … I am not frightened of falling again because you can’t go through your life being frightened.”* Participant #9

During the focus group therapists identified an important point about how some people have forgotten that they couldn’t get themselves up unassisted, thus creating a barrier to perceived need. Without having enough self-reflection to realise that getting up skills are needed, it is possible that any training may seem irrelevant to some patients.

*“They say … I can get up myself it’s absolutely fine I am independent … you speak to family … it comes out … they can’t get up. I am not sure of the reasons you know sometimes there’s a cognitive impairment and people don’t remember that they can’t get up. Family members tell me before that you know that dad’s, dad has a lot of pride he’s not going to tell you if he can’t do something”* Therapist focus group

### Decision context

The decision context encapsulated the many considerations that older people, their spouses and professionals balance when deciding upon actions to take. The reality is that there are far too many variables involved to always plan in advance, however the having the ‘know how’ on how best to get up and balancing if or when this might be appropriate, are key aspects of decision making.

#### Having the ‘know-how’

The value of training information was illustrated by one participants’ spouse, who had taken a different approach in their response when her husband fell out of a chair during the early morning. She initially checked that he was unhurt, then after being unable to seek her usual support from neighbours, she chose to search social media for advice on how to get up following a fall. She was able to successfully talk him through the process after watching an online video. For her this was a common way to find out how to learn new skills. She was the only participant who took this approach.

*“I thought well how do I get you up?...So, I thought let’s go onto YouTube … and looked it up and I said right you’ve got to get up on your knees and crawl to the chair or I might have put a stool or a kitchen chair there for him to use and get up. He said I can’t, I said you’re going to have to … Yeah, you’ve got to do it, and he did do it.”* Participant #12 spouse

For ambulance service staff training, there was specific information on the use of lifting equipment as and when new versions of training were introduced, however knowledge of recognised techniques for individuals to get themselves up was limited. This knowledge gap was recognised by one.

*“I think if as a paramedic I had some more skills in how to encourage people to get off the floor, that that would be useful.”* Ambulance service staff #4

Skills training is one aspect of the challenges staff face in adopting a more pro-active approach to teaching people to help themselves up from the floor, but perhaps a more difficult obstacle to overcome is that of cultural mind-set of reacting to the immediate problem in a way that fosters dependence rather than being able to encourage independence and develop getting up skills for people to utilise with any future falls.

*“'I must admit, one thing that I am aware of is the potential for patients to help themselves up in some situations. I think I quite often in the past worked on the assumption that if they were easily able to do that then they probably would have done it already, although that isn’t probably a very good assumption to make because I think people quite often don’t move because they’ve been advised not to move, rather than because they are unable to move. So, … I would say that I’ve been relatively quick to give people help to get up... and thinking about it now, I think I have been too ready to do that rather than trying to find ways that I could encourage them or support them to get themselves off the floor.”* Ambulance service staff #4

“*We see the people who are more frail and more vulnerable for the most part - we see people at the top of the pyramid, whereas those who would probably benefit most from the get up off the floor training are likely to be people who aren’t coming to our services. We might need to think more creatively about how we provide that training for them.”* Therapist focus group.

#### A balancing act

Both therapist and ambulance service staff alluded to the tension that they felt in balance professional responsibilities against each patients’ best interests. That balance was articulated in time pressures, awareness of short- and long-term benefits and tailoring information to the current needs of each person. Therapists often described longer opportunities to build their therapeutic relationship with their patients, but nevertheless, only able to offer a time-limited intervention. Ambulance service staff related how their ‘emergency’ response could also be an opportunity to check the patients’ home setting more holistically, see how they were looking after themselves, ask how they preferred help, offering encouragement to try other options to get themselves up so that they built personal confidence.

All health professionals recognised the physical and psychological damage that a ‘long lie’ could induce; ambulance service staff were keen to help people up quickly to limit that damage; therapists were sometimes hampered by patients’ psychological distress and fear following a previous fall, leading to an unwillingness to undergo training to learn how to get up from the floor.

*“I think when they’ve had a long lie [before being able to get up] that can have a massive psychological impact on their fear of moving forwards.”* Therapist focus group

Therapists recognised that earlier, proactive intervention could offer far more potential than their current response mode. Interestingly, we felt this was reflected in comments from a number of older people who spoke less of the techniques to get up, and more about needing to be ‘picked up’. A therapist suggests how timing of an intervention is important:

*“We’re talking about reaching a group when they have already had a fall, and there’s probably a group of people … who could benefit from that information before they are going to have that first fall … timing to implement change for people getting them to take on information is really, really crucial.”* Therapist focus group

And getting the timing post-fall correct:

*“The rehab stage … isn’t the time to be putting them on the floor because they’re actually recovering … but maybe three months down the line then that could be practised with them to really consolidate that information but it’s the timing”* Therapist focus group

The ambulance service staff did not refer to terminology commonly associated with getting up techniques, their focus was more on immediate threats to life and health to minimise danger, to initiate referrals to ensure appropriate services would be put in place, and to ensure efficient use of services.

*“The first priority was to get to the person and deal with them in a timely fashion and the second, sort of, benefit hopefully would be that we wouldn’t need an ambulance so we could keep an ambulance on the road that could attend somebody who was in dire need”* Ambulance service staff #1

*“As soon as I’ve got them at the point where they are telling me that they are not in pain then I’ll try and move them straightaway.”* Ambulance service staff #3

Many of the healthcare professionals mentioned the need to do things differently from just being reactive services to also incorporate more proactive roles by recognising and working with the person’s potential within an appropriate timeframe. Participants and their carers showed less awareness of getting up techniques or training opportunities but felt frustrated or fearful following a fall that they could not get themselves up from, and expressed reluctance to have to call on others for help.

## Discussion

### Summary

We investigated help-seeking and the use of techniques to get up following a fall; we identified three main areas that impacted on capability and should form the basis of any interventions: physical ability, self-efficacy, and the physical and social environment. The decision context comprises of having the know-how on how to best get up and the balancing act of if and when to intervene.

### Comparison with existing literature

To our knowledge this is this first qualitative study exploring attitudes towards older people getting up following a fall incorporating perspectives of older people, their spouses, and healthcare professionals. Most falls research focuses on prevention, particularly improving balance and reducing environmental hazards, but there is little attention paid to managing the fall itself [18]. From the evidence on falls prevention, the model of potential intervention considerations we developed (Fig. 1) is also supported by existing quantitative and qualitative evidence [8, 11, 21]. Physical and functional impairments [22] and living alone are associated with ability to get up after a fall [23]; only one staff participant highlighted that time and exercise training may be required to get people to the point of having the physical ability to get up, yet the benefits of maintaining activity for older people is recognised and has similar barriers to those we identified. In common with the recent study by Maula and colleagues (2019) [24], we also found that novel use of technology, in the form of on-line information, was used within our sample, applied in such a way that ongoing knowledge and behaviour was changed after accessing on-line guidance.

Fear of falling is present on 59% of those who have fallen and 40% of those who haven’t fallen [25]. A study of over 16,000 older people living in Canada suggested that most injurious falls were not associated with fear of falling and that older women who experienced very severe fall injuries had an increased odds of fear of falling [26]. Evidence from people with Parkinson’s disease suggests that different strategies to handle fear of falling are adopted, with the experience of fear being complex and varying over time and in different environments [21]. Our data corroborates the importance of the fear of falling in peoples’ willingness to try to learn new techniques to help themselves up. Addressing the relationship between fear, self-efficacy and ability to get up is key in developing individuals’ capability to manage a falls response themselves. In our sample the inability to get up was particularly concerning to people in their outdoor environment. It has been suggested those who experience injurious falls or need medical help as a result of falling are more likely to have a fear of falling outdoors [25]. The characteristics of outdoor falls are known to engender feelings of embarrassment and anxiety about future falls [27] and Cox and Williams (2016) suggest fear of falling may be based on a fear of inability to get up, rather than of the fall itself [28]. This would support a wider dissemination of these skills to reduce ongoing anxiety about getting up from an outdoor fall. A recent study found that fear of falling was strongly associated with dependence in activities of daily living to a greater extent than falls or injuries and that rehabilitation programmes should ensure management of fear is an integral part [29]. Our findings provide a valuable addition to the existing literature on fear of falling and rehabilitation to promote independence for those who have fallen or had fall-related injuries, as well as enabling those who haven’t fallen to maintain their independence.

Only one formal technique for teaching how to get up was mentioned by the healthcare professionals; this being ‘backward-chaining’ [17]. It was developed to minimise the likelihood of failure by breaking the activity down into small steps, with the final step being taught first. The approach reduces the anxiety of both the physiotherapists teaching and the person being taught when compared with a conventional technique where the person has to be able to get completely down onto the floor before they are taught to get up [18]. It has been reported to be acceptable to patients and more successful than using other approaches [17]. All the therapists were knowledgeable about the technique; however, the ambulance service staff were not. Although the latter have a remit for emergency care, some reflected that receiving training could enable them to have a more holistic approach to patient care where time allowed. The increasing numbers of ambulance service staff working in primary care could potentially be a key group to train. We found that therapists were also keen to broaden and utilise their skills to operate a more preventative ‘healthy ageing’ role, by ensuring older people are able to get up rather than have to retrain when physical ability has gradually reduced over time. Effective translation of our findings into practice will require targeted training that is meaningful, feasible and motivates people to put new training into practice [30].

### Strengths and limitations

The study generated rich data from different perspectives, and we had a diverse range of older participants in terms of age, sex and living circumstances, and health care professionals in terms of professional background, grade, sex and work locations in South West England. This said, the older participants who took part in the study all had recent contact with falls or rehabilitation services with no recruitment from the ambulance service. This was likely due to users of the ambulance service having more acute needs and therefore staff may have felt that it was inappropriate to approach people to take part in a study. In addition, there was a lack of ethnic diversity amongst the participants. This reflects our regional population but may limit the application of our findings to other communities.

### Implications for research and practice

Our research provides a valuable addition to the small body of work on managing falls amongst older people, however it remains unknown whether improving people’s ability to get up following a fall (a) would impact on ambulance callouts and (b) the cost effectiveness of such an intervention.

Although teaching how to get up from a fall is recommended for those at risk of falling [12] there is often limited therapist capacity to be able to do this, therefore training and information could also be provided to others who live or work with older people to ensure they also have the ‘know how’. As well as ambulance service staff this could also include other groups such as exercise professionals, care workers and informal carers, and other healthcare professionals working with older people. In addition, resources for older people and their families to enable them to practice getting up, such as the film produced by NHS Inform in Scotland [31], could be a valuable supplement to face-to-face training or as a self-management resource. However, future research is required to establish whether undertaking ‘getting up’ training is effective and cost-effective at, for example, reducing the consequences of a long lie or ambulance callouts.

## Conclusion

We identified how this cohort of older people, carers and healthcare professionals decide what to do after a fall. People’s capability to get up after a fall is affected by a combination of physical ability, self-efficacy and their physical and social environment within a decision context that encapsulates knowing how to get up, and balancing if or when this might be appropriate. Healthcare professionals have to respond to current need but recognize the value of a more proactive approach to manage future falls. Teaching interventions to change the nature of individuals’ response to a fall could be beneficial in terms of improving personal independence and better managing the use of NHS resources.

## Supplementary information

**Additional file 1.** Interview topic guide.

## Data Availability

The datasets generated and/or analysed during the current study are not publicly available due to privacy concerns but are available from the corresponding author upon reasonable request.

## Abbreviations

UK: United Kingdom
NHS: National health service

## References

[1] Public Health England. Falls: applying All Our Health. 2020. https://www.gov.uk/government/publications/falls-applying-all-our-health/falls-applying-all-our-health#further-reading-resources-and-good-practice. Accessed 31 Jan 2020.

[2] Hopewell S, Adedire O, Copsey BJ, Boniface GJ, Sherrington C, Clemson L, Close JC, Lamb SE (2018). Multifactorial and multiple component interventions for preventing falls in older people living in the community. Cochrane Database Syst Rev.

[3] Sherrington C, Fairhall NJ, Wallbank GK, Tiedemann A, Michaleff ZA, Howard K, Clemson L, Hopewell S, Lamb SE. Exercise for preventing falls in older people living in the community. Cochrane Database Syst Rev. 2019;1. 10.1002/14651858.CD012424.pub2 PMC6360922 30703272

[4] Cameron ID, Dyer SM, Panagoda CE, Murray GR, Hill KD, Cumming RG, Kerse N. Interventions for preventing falls in older people in care facilities and hospitals. Cochrane Database Syst Rev. 2018;9. 10.1002/14651858.CD005465.pub4 PMC6148705 30191554

[5] Chalk D, Black S, Mitt M (2016). Which factors most influence demand for ambulances in the south west?. J Paramed Pract.

[6] O'Cathain A, Jacques R, Stone T, Turner J (2018). Why do ambulance services have different non-transport rates? A national cross sectional study. PLoS One.

[7] Jorstad-Stein EC, Hauer K, Becker C, Bonnefoy M, Nakash RA, Skelton DA, Lamb SE (2005). Suitability of physical activity questionnaires for older adults in fall-prevention trials: a systematic review. J Aging Phys Act.

[8] Fleming J, Brayne C. Inability to get up after falling, subsequent time on floor, and summoning help: prospective cohort study in people over 90. BMJ. 2008;337. 10.1136/bmj.a2227 PMC2590903 19015185

[9] Ebben RHA, Vloet LCM, Speijers RF, Tonjes NW, Loef J, Pelgrim T, Hoogeveen M, Berben SAA (2017). A patient-safety and professional perspective on non-conveyance in ambulance care: a systematic review. Scand J Trauma Resusc Emerg Med.

[10] Davey C, Wiles R, Ashburn A, Murphy C (2004). Falling in Parkinson's disease: the impact on informal caregivers. Disabil Rehabil.

[11] Ang SGM, O'Brien AP, Wilson A (2019). Understanding carers' fall concern and their management of fall risk among older people at home. BMC Geriatr.

[12] Goodwin V, Briggs L (2012). Guidelines for the physiotherapy management of older people at risk of falling.

[13] Lamb S, Gates S, Fisher J, Cooke M, Carter Y, McCabe C (2008). Scoping exercise on fallers' clinics: report to the National Coordinating Centre for NHS service delivery and organisation (NCCSDO).

[14] Goodwin V, Martin FC, Husk J, Lowe D, Grant R, Potter J (2010). The national clinical audit of falls and bone health - secondary prevention of falls and fractures: a physiotherapy perspective. Physiotherapy.

[15] Dean SG, Leon P, Warmoth K, Goodwin VA, Stiles VH, Taylor RS (2019). Independently getting off the floor: a feasibility study of teaching people with stroke to get up after a fall. Int J Ther Rehabil.

[16] Burton E, Farrier K, Lewin G, Petrich M, Boyle E, Hill KD. Are interventions effective in improving the ability of older adults to rise from the floor independently? A mixed method systematic review. Disabil Rehabil. 2020;42(6):743–53. 10.1080/09638288.2018.1508509 30512983

[17] Leonhardt R, Becker C, Gross M, Mikolaizak AS. Impact of the backward chaining method on physical and psychological outcome measures in older adults at risk of falling: a systematic review. Aging Clin Exp Res. 2020;32(6):985–97. 10.1007/s40520-019-01459-1 31939202

[18] Reece AC, Simpson JM (1996). Preparing older people to cope after a fall. Physiotherapy.

[19] Saunders B, Sim J, Kingstone T, Baker S, Waterfield J, Bartlam B, Burroughs H, Jinks C (2018). Saturation in qualitative research: exploring its conceptualization and operationalization. Qual Quant.

[20] Gale NK, Heath G, Cameron E, Rashid S, Redwood S (2013). Using the framework method for the analysis of qualitative data in multi-disciplinary health research. BMC Med Res Methodol.

[21] Jonasson SB, Nilsson MH, Lexell J, Carlsson G (2018). Experiences of fear of falling in persons with Parkinson's disease - a qualitative study. BMC Geriatr.

[22] Schwickert L, Oberle C, Becker C, Lindemann U, Klenk J, Schwenk M, Bourke A, Zijlstra W (2016). Model development to study strategies of younger and older adults getting up from the floor. Aging Clin Exp Res.

[23] Bergland A, Laake K (2005). Concurrent and predictive validity of "getting up from lying on the floor". Aging Clin Exp Res.

[24] Maula A, LaFond N, Orton E, Iliffe S, Audsley S, Vedhara K, Kendrick D (2019). Use it or lose it: a qualitative study of the maintenance of physical activity in older adults. BMC Geriatr.

[25] Chippendale T, Lee CD (2018). Characteristics and fall experiences of older adults with and without fear of falling outdoors. Aging Ment Health.

[26] LeBouthillier DM, Thibodeau MA, Asmundson GJ (2013). Severity of fall-based injuries, fear of falling, and activity restriction: sex differences in a population-based sample of older Canadian adults. J Aging Health.

[27] Nyman SR, Ballinger C, Phillips JE, Newton R (2013). Characteristics of outdoor falls among older people: a qualitative study. BMC Geriatr.

[28] Cox TB, Williams K (2016). Fall recovery intervention and its effect on fear of falling in older adults. Act Adapt Aging.

[29] Pereira C, Bravo J, Raimundo A, Tomas-Carus P, Mendes F, Baptista F. Risk for physical dependence in community-dwelling older adults: The role of fear of falling, falls and fall-related injuries. Int J Older People Nurs. 2020;15(3):e12310. 10.1111/opn.12310 32083403

[30] Hill K (2009). Don't lose sight of the importance of the individual in effective falls prevention interventions. BMC Geriatr.

[31] NHSinform. Upwards and Onwards. https://www.nhsinform.scot/healthy-living/preventing-falls/dealing-with-a-fall/what-to-do-if-you-fall. Accessed 16 Sept 2020.

