# Supplementary material for: Knowledge, skills and attitudes of older people and staff about getting up from the floor following a fall: a qualitative investigation
Source: BMC Geriatr. 2020 Oct 6;20:385. doi: 10.1186/s12877-020-01790-7 (PMC7542342; doi:10.1186/s12877-020-01790-7)
Supplement: Supplementary file 1 — Additional file 1. Interview topic guide. [file 12877_2020_1790_MOESM1_ESM.docx]

**‘Getting Up’ Interview Topic Guide**

Introductions and confirmation of consent process and voluntary nature of the research.

*The purpose of today’s interview is to find out about how you (or a companion if you were not alone) made a decision about how to seek help following your fall. We’re particularly interested in how you (or your companion) decided whether to call for assistance or not or what you did if you didn’t call for help.*

Note general background details: age, general health status, history of falling.

*I’m going to start by asking you to think back to when you found yourself on the floor and talk me through what you can remember about making a decision about what to do. I’ll move on to ask about what happened next e.g. if called an ambulance or other help then once the ambulance arrived, how long they stayed, who else they spoke to, then finish with any thoughts you have about what you would do if it happened again or any times when you fell and did things differently (were able to get up or get different help).*

**1. The fall**

*Thinking back to the day you had a fall, what do you remember from the moment you fell onwards?*

Prompts: When it happened, where, if it happened before, what happened last time, was anyone with you, how were you feeling, how was your companion (if anyone else was there) feeling?

**2. Deciding what to do**

*Moving on to deciding if you needed help once you were on the floor, can you talk me through how you decided what to do next?*

Prompts: Were you conscious all the time? Had you tried to move or get up, yourself or with help? How did that feel? What were your (or their if companion present) main concerns?

**2. Calling for assistance (the ambulance or family)**

*Did you call for assistance? Who made the phone call and what you or they were thinking when the call was made?*

Prompts: How long do you think you’d been on the floor when (if) the ambulance was called? What did you think about going to hospital? How long were you told you would expect to wait?

**3. Once help arrived**

*Once help arrived can you tell me what happened?*

Prompts: Who arrived, how many, how long did they take to arrive, who did they speak to, how did you get up? How did you feel about not having to go to hospital? Were you given any advice about what to do if it happened again? Did you need any type of follow-up? Maybe from your GP?

**4. Is there anything you feel might change what you did after a fall?**

*Thinking about the future, if you had a similar fall next month is there anything you would consider doing differently?*

Prompts: Is there anything that you might consider instead of calling an ambulance or assistance? What sort of things might help you to get up without help? Maybe information or advice? Are there any other services you might consider calling?

**‘Getting Up’ Focus Groups: Topic Guide for ambulance service staff**

Introductions & re-iterate the voluntary nature of participation.

*The purpose of this group today is to find out more about how decisions with patients are made about getting up from the floor following a fall. We are most interested in patients who fall but are uninjured. We’re interested in how and why they call for help getting up off the floor, and any information or training that staff find helpful in preparing patients to tackle decisions after a fall for themselves.*

*I’d like to start with your experience of responding to a patient’s fall and hear about how you work through the decision process about conveying to hospital or not, thinking particularly about the getting up from the floor stage. I’ll move on to ask more generally about how you link with other services, then conclude with some reflection on what you think is best about current care and what could make it better.*

1. **Deciding if transport to hospital is necessary**

*So thinking about when you believe the patient has had a fall but sustained no substantial injury from it, how do you work through the decision about whether to convey a patient to hospital or not?*

Prompts: How does what the patient *says* influence you? How does what the patient *does* influence you? Maybe how they’re able to get up? How different is it when a companion is present? How is your choice influenced by service guidelines? How important is timing (time of day, length of time on the floor)?

1. **Linking with other services**

*Could you explain how any other health services or personnel feed into your decision not to convey a patient to hospital?*

Prompts: Do you call any other health professional when making decision about non-conveyance (such as the GP or the hub)? How does this call influence your decision making? How often does this happen? Which other services link into that decision?

1. **When leaving the patient**

*Thinking about the patient again, when you leave them are there any follow-up actions?*

Prompts: What advice do you leave that patient with? How do you decide whether to send a report to the GP or other HCP about the attendance?

*For a person who experiences recurrent falls, so perhaps you have seen a number of occasions, is there anything you do differently?*

1. **Current and future services**

*Thinking about current services what would you say is best about how services support patients who have a fall now?*

Prompts: Speed of response, types of interventions, follow-up arrangements.

*Thinking about how we might do it better in the future, what do you think could support your patients following a fall instead of what happens now?*

Close, thanks, and *is there anything else you think important about supporting patients to safely get themselves up after a fall that we haven’t talked about?*

**‘Getting Up’ Focus Groups: Topic Guide for Therapists**

Introductions & re-iterate the voluntary nature of participation.

*The purpose of this group today is to find out more about how decisions with patients are made about getting up from the floor following a fall. This is not about falls prevention which you’ll all be aware of, but about decision making after a fall. We are most interested in patients who fall but are uninjured. We’re interested in how and why they call for help getting up off the floor, and any information or training that staff find helpful in preparing patients to tackle decisions after a fall for themselves.*

*I’d like to start with your experience of your patients falls risk and ability to respond after a fall. I’ll move on to ask more about the type of training you’ve received and advice you give your patient about getting up or moving around after falling over. I’d like to finish with your thoughts on current and future service provision.*

1. **The scale of falls, number at risk and their ability to respond**

*Can we start with how many of your patients have falls?*

Prompts: How many of your caseload? How many are at risk of falling? Of those how many would you say were able to get themselves up off the floor?

1. **Communication with patients and teaching them to get up**

*Thinking about how you tackle the topic of falls with your patients, how much would you say you talk with your patients about getting up following a fall?*

Prompts: How do you discuss what to do if they fall? How many of your patients do you teach how to get themselves up from the floor following a fall? How do you choose which ones to teach this to? Do you give them any written advice about getting up from the floor?

1. **Skills and training**

*What skills would you say a patient needs to be able to get up from the floor?*

Prompts: level of strength, ability to move, how important is confidence?

*What training or support do you have about teaching patients to get up from the floor?*

Prompts: When did you get that training? What was it part of? How (if at all) did it affect your practice?

What contact or support do you have with other services about patients who have a fall?

1. **Current and future services**

*Thinking about current services what would you say is best about how services support patients who have a fall now?*

Prompts: Types of interventions, follow-up arrangements. What barriers do you see to teaching all of your patients how to get up from the floor following a fall? And what might facilitate that to become routine?

*Thinking about how we might do it better in the future, what do you think could support your patients following a fall instead of what happens now?*

Close, thanks, and *is there anything else you think important about supporting patients to safely get themselves up after a fall that we haven’t talked about?*
